# Granite Extraction Based on the SDGSAT-1 Satellite Thermal Infrared Spectrometer Imagery

**DOI:** 10.3390/s24061750

**Published:** 2024-03-08

**Authors:** Boqi Yuan, Qinjun Wang, Jingyi Yang, Wentao Xu, Chaokang He

**Affiliations:** 1 Key Laboratory of Digital Earth Science, Aerospace Information Research Institute, Chinese Academy of Sciences, Beijing 100094, China; yuanboqi23@mails.ucas.ac.cn (B.Y.); yangjingyi211@mails.ucas.ac.cn (J.Y.); xuwentao221@mails.ucas.ac.cn (W.X.); hechaokang8311@163.com (C.H.); 2 International Research Center of Big Data for Sustainable Development Goals, Beijing 100094, China; 3 College of Resources and Environment, Yanqi Lake Campus of University of Chinese Academy of Sciences, Beijing 101408, China; 4 Kashi Aerospace Information Research Institute, Kashi 844199, China; 5 Key Laboratory of the Earth Observation of Hainan Province, Hainan Aerospace Information Research Institute, Sanya 572029, China; 6 College of Geoscience and Surveying Engineering, China University of Mining & Technology (Beijing), Beijing 100083, China

**Keywords:** Karamay, SDGSAT-1 TIS, granite extraction, band ratio, Sauvola threshold

## Abstract

Earth observation by remote sensing plays a crucial role in granite extraction, and many current studies use thermal infrared data from sensors such as ASTER. The challenge lies in the low spatial resolution of these satellites, hindering precise rock type identification. A breakthrough emerges with the Thermal Infrared Spectrometer (TIS) on the Sustainable Development Science Satellite 1 (SDGSAT-1) launched by the Chinese Academy of Sciences. With an exceptional 30 m spatial resolution, SDGSAT-1 TIS opens avenues for accurate granite extraction using remote sensing. This study, exemplified in Xinjiang’s Karamay region, introduces the BR-ISauvola method, leveraging SDGSAT-1 TIS data. The approach combines band ratio with adaptive k-value selection using local grayscale statistical features for Sauvola thresholding. Focused on large-scale granite extraction, results show F1 scores above 70% for Otsu, Sauvola, and BR-ISauvola. Notably, BR-ISauvola achieves the highest accuracy at 82.11%, surpassing Otsu and Sauvola by 9.62% and 0.34%, respectively. This underscores the potential of SDGSAT-1 TIS data as a valuable resource for granite extraction. The proposed method efficiently utilizes spectral information, presenting a novel approach for rapid granite extraction using remote sensing TIS imagery, even in scenarios with low spectral resolution and a single data source.

## 1. Introduction

Granite is an intrusive igneous rock formed by the cooling and solidification of magma deep in the Earth’s crust, containing abundant mineral elements. Its formation is typically associated with geological processes such as hydrothermal activities and metasomatism, and is accompanied by tectonic movements, providing direct conditions for the formation of mineral deposits [1]. Additionally, granite can also serve as a crucial raw material in the fields of construction and manufacturing. The traditional identification of granite relies on field exploration; however, remote sensing technology, with its wide coverage and dynamic monitoring capabilities, greatly reduces the workload in the field [2]. Therefore, the extraction of granite information from suitable remote sensing images is of significant importance for mineral resource exploration, geological evolution studies, and geological surveys.

Due to differences in compositional and structural characteristics, all rock minerals exhibit distinct spectral features in remote sensing images. To date, many scholars have conducted research on lithology extraction based on this principle, roughly categorized into lithology extraction based on image enhancement techniques and classification based on machine learning methods [3,4,5,6,7,8,9]. Image enhancement techniques commonly employ classic methods such as Landsat, ASTER, and Sentinel-2 data, combined with techniques like principal component analysis (PCA), band ratio (BR), matched filtering (MF), and color composite (CC) [10,11,12,13]. These methods often require human–machine interaction for interpretation. In machine learning classification, the Support Vector Machine (SVM) classifier has achieved tremendous success [9,14]. However, image segmentation techniques are rarely adopted in lithology extraction research. Among them, the Sauvola threshold segmentation method shows tolerance to lighting variations and image noise in different regions and is relatively easy to implement [15,16]. The Western Junggar region is located in a crucial position in the southwestern part of the Central Asian orogenic belt, with highly developed Late Paleozoic magmatic rocks. Common rock-forming minerals such as silicate and carbonate minerals exhibit obvious emission spectra in the thermal infrared bands, making lithology extraction through remote sensing relatively straightforward [17,18].

Although the above research has proven to be effective in lithology extraction, the following challenges still exist. (1) Spatial resolution limitations: the commonly used ASTER, Landsat, and other thermal infrared data have spatial resolutions of about 100 m, which cannot provide precise spatial information. On the other hand, acquiring high-spectral and high-resolution data involves significant costs, and these datasets often have limited coverage. (2) Complex processes: The process of manual interpretation, feature selection, and model training is labor-intensive and time-consuming, making it challenging to achieve rapid extraction of granitic lithology [19]. There is an urgent need for the development of a fast and efficient method for granite lithology extraction.

The Sustainable Development Science Satellite-1 (SDGSAT-1), launched on November 5, 2021, and equipped with a multispectral, glimmer, and thermal infrared imager, is China’s first satellite dedicated to serving the United Nations’ 2030 Sustainable Development Agenda. Notably, the thermal infrared sensor possesses imaging capabilities with a 300 km swath and a spatial resolution of 30 m, addressing the low spatial resolution drawback in the thermal infrared spectral range of multispectral sensors. Scholars have used its data to conduct studies related to thermal emissions, but no studies have been found for lithology extraction [20,21]. In response to the current research gap, this paper proposes a method for extracting granite based on SDGSAT-1 TIS data, whereby a granite index based on SDGSAT-1 TIS data is first introduced using band ratio to enhance granite and suppress non-target information. Then, an improved Sauvola algorithm, ISauvola, is applied to the image after band ratio operation (BR) to achieve rapid and efficient extraction of granite from the SDGSAT-1 TIS data.

## 2. Methods

### 2.1. Flowchart of the Method

**Figure 1:**
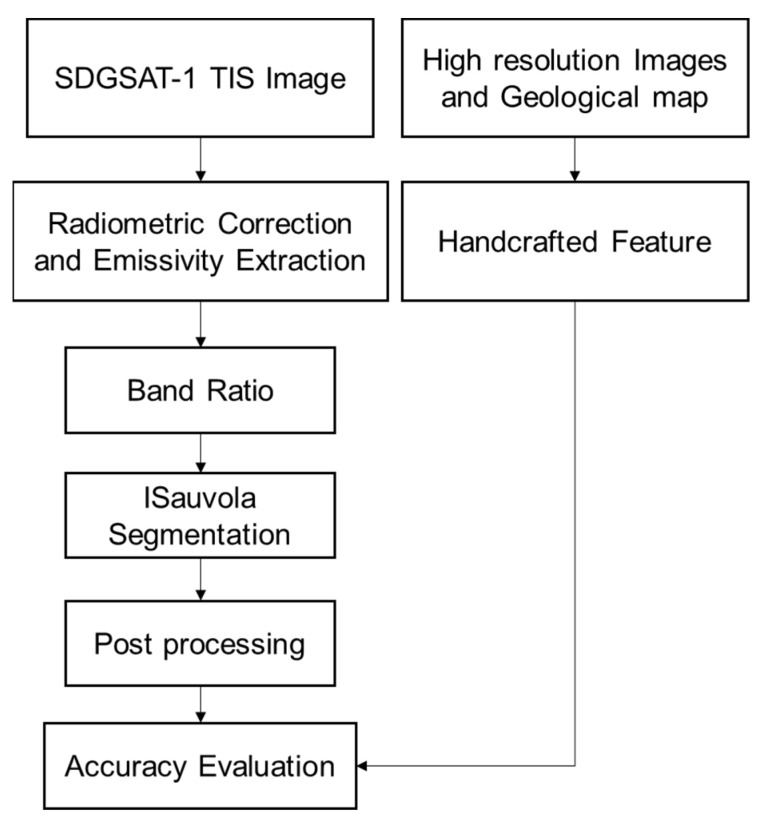
Flowchart of the granite extraction method.

The technical flowchart of this study is shown in Figure 1. Firstly, SDGSAT-1 TIS data were acquired and preprocessed, including temperature and emissivity separation (TES), to obtain the surface emissivity map of the study area. Then, a granite index (GI) was established based on the characteristics of the thermal infrared emission spectral profile of granite for band ratio operation, followed by an improved Sauvola threshold segmentation traversing the image to compute the threshold value. Then, the final granite region was delineated by post-processing, such as block elimination and hole filling. Finally, a precision assessment of the granite extraction results is conducted using precision, recall, and F1 score.

### 2.2. Band Ratio

**Figure 2:**
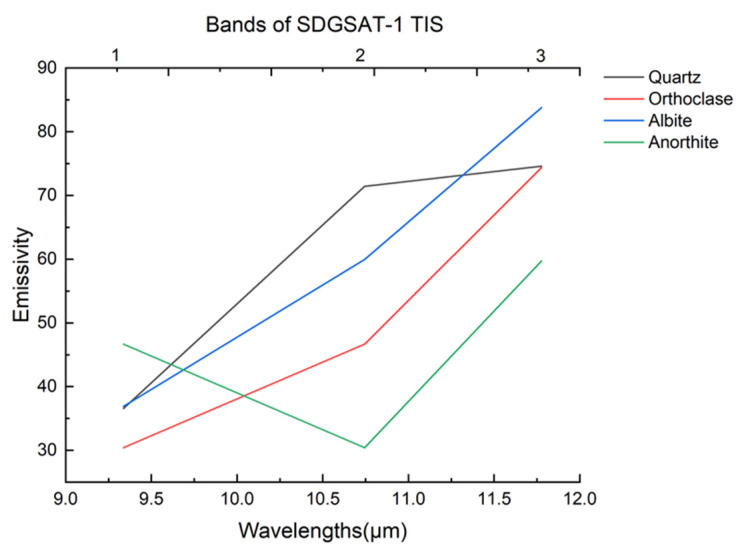
Thermal infrared spectra of granitic rock-forming minerals resampled to the SDGSAT-1 TIS band passes.

Band ratio is a commonly used technique in remote sensing image processing, where the ratio between different bands of an image is calculated to highlight the absorption characteristics of target features. Simultaneously, it can suppress the influence of brightness differences in the image, such as topographic shadows and sensor noises. In lithological identifications, Ninomiya et al. established various mineral indices, such as the Quartz Index (QI), based on ASTER TIR data [22]. Granite, which is primarily composed of quartz and feldspar, exhibits distinct thermal infrared emission spectra as shown in Figure 2. These minerals show low emission at 8–12 μm bands due to the vibration of SiO4 groups. Specifically, quartz presents a low emission peak at 9.25 μm, while feldspar displays a continuous low emission at 8.7–9.8 μm, corresponding to band 1 of SDGSAT-1 TIS data. After 10μm, their emissivity gradually increases. Based on these characteristics, the Granite Index (GI) for SDGSAT-1 TIS data is formulated as follows (the correspondence between the sensor bands and wavelengths is shown in Table 1):

(1) $$\mathrm{GI}=\frac{B_{2}\times B_{3}}{{B_{1}}^{2}}$$

### 2.3. Improved Sauvola Segmentation (ISauvola)

Traditional threshold segmentation usually selects a threshold to divide the whole image into two values. For example, the most commonly used Otsu method can automatically calculate the best global threshold value, but the global threshold segmentation has poor performance on images with uneven illumination and gray values. To solve this problem, we adopt the local segmentation method for processing, of which the most representative one is the Sauvola algorithm, which has an excellent performance in image extraction. It calculates the segmentation threshold of the pixel by calculating the mean and variance of pixel gray level in the neighbor pixels and has good adaptability to noises. The formula is shown as follows:

(2) $$T\left(i,\;j\right)=m(i,j)\left[1+k(\frac{s\left(i,j\right)}{R}-1)\right]$$

where $T\left(i,j\right)$ represents the threshold of pixel (i,j); m(i,j) and s(i,j) are the mean and standard deviation values of the pixel (i,j)’s neighborhood, respectively. k is an adjustable parameter with a range of [0, 1], and R is the dynamic range of the standard deviation, which is 128 for an 8-bit grayscale image.

Taking an 8-bit image as an example, when k ∈ [0, 1], the value of $s\left(i,j\right)$ is always less than or equal to 128. Consequently, the calculated threshold is always less than or equal to m(i,j), and the selection of the threshold is highly influenced by the adjustable parameter [15]. In theory, when the window brightness is high, the threshold should be relatively high. Therefore, the parameter k should be appropriately small (since this paper extracts highlighted areas in the image, the range of k is adjusted accordingly to [−1, 0], and in such case, $T\left(i,j\right)$ is constantly greater than or equal to m(i,j)), reducing the likelihood of misclassifying the background as the target. When the window brightness is low, the threshold should be relatively small. The parameter k should be appropriately increased to avoid losing the target. Thus, a relationship can be established between k and the statistical values of window pixels. Additionally, unlike text images, the grayscale heterogeneity between windows in this image is significant. Using a fixed R-value cannot reflect this difference adequately. Therefore, the standard deviation coefficient R is replaced with the difference between the maximum and minimum values. This modification ensures a relatively stable calculation of the threshold. In summary, the analysis leads to

(3) $$T\left(i,\;j\right)=m\left(i,j\right)\left[1+k\left(\frac{s\left(i,j\right)}{K_{\max}-\;K_{\min}}-1\right)\right]$$

where

(4) $$k=\frac{\sqrt{m\left(i,j\right)}}{R}$$

Equations (3) and (4) establish the relationship between k, local threshold $T\left(i,j\right)$, and local pixel statistics. When the local brightness is high, it enables an adaptive increase in the threshold, and conversely, a decrease. This adaptation is better suited for granite extraction.

### 2.4. Accuracy Evaluation

The accuracy assessment is based on a confusion matrix calculated using predicted and true values. Precision, recall, and F1 score are the metrics employed to evaluate the accuracy of granite extraction.

Precision is the proportion of true positive samples (predicted as 1 and actually 1) among all samples predicted as 1, with the following formula:

(5) $$\mathrm{Precision}=\frac{\mathrm{TP}}{\mathrm{TP}+\mathrm{FP}}$$

where TP is the number of true positive samples where the prediction and the actual values are both positive, and FP is the number of false positive samples where the prediction is positive but the actual value is negative.

Recall is the proportion of true positive samples (predicted as 1 and actually 1) among all samples that are actually 1. It is calculated using the following formula:

(6) $$\mathrm{Recall}=\frac{\mathrm{TP}}{\mathrm{TP}+\mathrm{FN}}$$

where TP is the number of true positive samples where the prediction and the actual values are both positive, and FN is the number of false negative samples where the prediction is negative but the actual value is positive.

F1 score is defined as the harmonic mean of precision and recall. It is calculated by the formula

(7) $$F1=2\cdot \frac{\mathrm{precision}\cdot \mathrm{recall}}{\mathrm{precision}+\mathrm{recall}}$$

In this context, the predicted values are the results extracted using ISauvola, and the ground truth values are obtained through manual interpretation based on Google Earth Pro’s high-resolution images combined with 1:200,000 geological maps.

## 3. Experiment

### 3.1. Study Area

**Figure 3:**
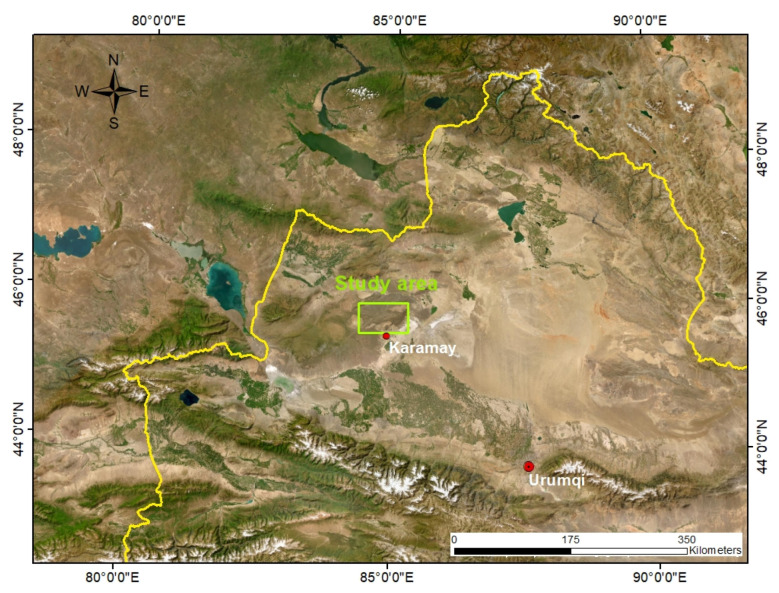
Geographical location of the study area. The yellow line is the national borders of China.

As shown in Figure 3, the study area is located in the Karamay Hinterland of West Junggar, belonging to the southwestern part of the Central Asian Orogenic Belt, with the geographical coordinates of 84°26′35″ to 85°17′30″ E and 45°37′06″ to 46°1′08″ N, covering an area of about 2900 km^2^. It experiences a temperate continental climate characterized by low rainfall and arid conditions throughout the year. The geological formations in the area can be broadly categorized into four periods. The most widespread deposits are the relatively thick Carboniferous volcanic clastic sedimentary rocks, including the Xibeikulasi, Baogutu, and Tailegula strata. These strata consist mainly of conglomerates, sandstones, siltstones, mudstones, siliceous rocks, and tuffaceous rocks [23,24]. Within these sedimentary strata, two Devonian ophiolite belts, the Darbut and Karamay belts, with an NE trend are exposed. Rock types within these belts include serpentinite, diabase, and basalt [25,26,27]. In addition, the region features extensive Late Carboniferous to Early Permian medium-acidic magmatic intrusions in the form of massive outcrops. The main rock types include alkaline granite, syenogranite, and granodiorite, constituting several intrusions such as Akbastao, Karamay, Xiaerpu, Hongshan, and Hatu, whose formation time is estimated to be 300 ± 20 Ma [26,28]. In the southeastern hills, there are Triassic sedimentary mudstones, while the lower-altitude plains in the east and southeast are mainly Quaternary alluvial and fluvial deposits, which are composed of sands and gravel.

### 3.2. Data and Preprocessing

We used a single scene of SDGSAT-1 TIS L4A-level imagery, named as KX10_TIS_20220404_E85.12_N46.72_202200093983_L4A. L4 products are orthorectified using GCPs and DEM based on L1 products, which are obtained by relative radiometric correction and RPC correction using the original images. The detailed sensor parameters are shown in Table 1. Specifically for its low cloud cover, this image was selected to facilitate the extraction of surface lithological information. The imagery underwent precise orthorectification using an advanced ground control point, eliminating the need for additional geometric and terrain corrections. The projection coordinate system for the image was WGS_1984_UTM_Zone_45N, and the acquisition date was 4 April 2022.

The preprocessing steps include radiometric calibration, atmospheric correction, and emissivity inversion. Firstly, the thermal infrared data are stacked, then its absolute radiance values (DN) are converted into reflectance values using the following formula based on the sensor calibration coefficients from Table 2:

(8) L=DN×Gain+Bias

where L represents the radiance at sensor (Wm^−2^ sr^−1^μm^−1^), and DN is the image digital number.

In this paper, we use the Thermal Atmospheric Correction and Emissivity Normalization modules of the ENVI 5.3 software to complete the atmospheric correction and emissivity inversion of the data. The atmospheric correction is used to calculate the surface emissivity, and the atmospherically corrected thermal infrared data are cropped to obtain the study area range. According to Planck’s law, if the emissivity ε(λ) is known, we can find the temperature of the target T and vice versa.

(9) $$\varepsilon \left(\lambda \right)=\frac{L\left(\lambda ,T\right)}{B\left(\lambda ,T\right)}=\frac{\pi \lambda^{5}\left(e^{\frac{c_{2}}{\lambda T}}-1\right)L\left(\lambda ,T\right)}{c_{1}}$$

where λ is the measured wavelength; L(λ, T) is the measured value; and c1, c2, and π are constants.

Emissivity normalization, which has been validated as one of the accurate methods for emissivity extraction, is an improvement on the reference channel method and applicable to any thermal infrared dataset [29,30].

### 3.3. Results and Analysis

**Figure 4:**
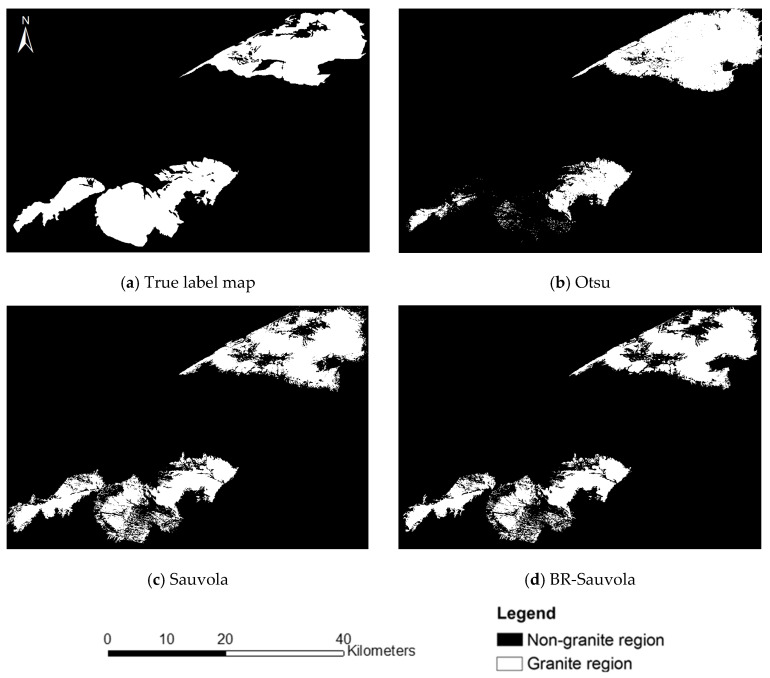
Extracted granite map using different methods.

**Figure 5:**
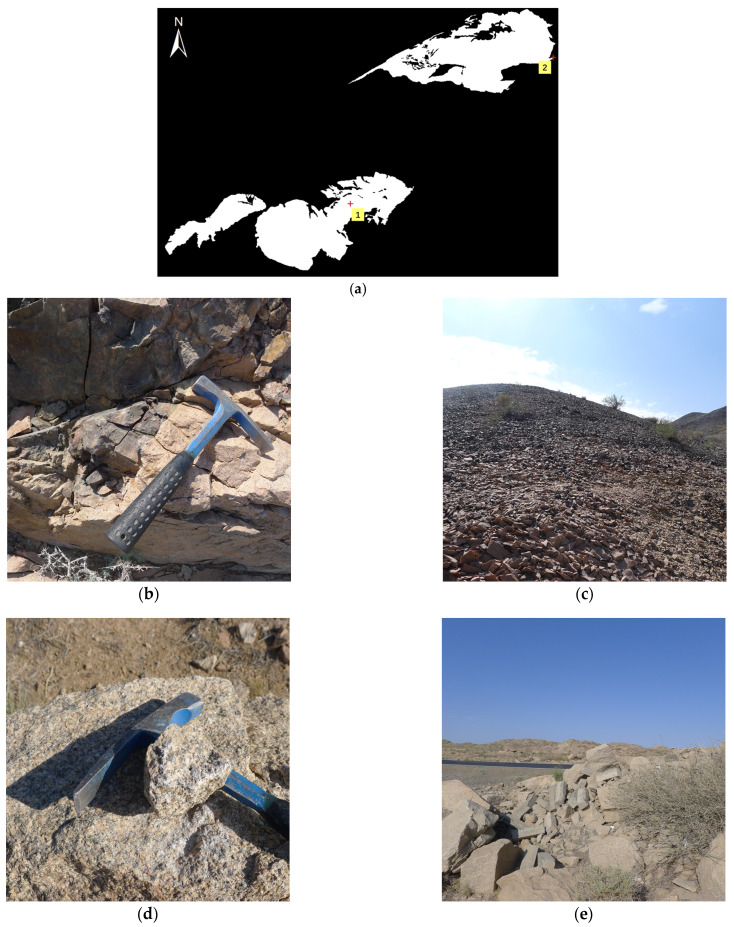
Field validation results of granitic lithology extraction in Karamay. (**a**) Point 1, 2 are located in Karamay and Hongshan plutons respectively, denoted by red plus sign. (**b**,**c**) Field view of point 1 in (**a**). (**d**,**e**) Field view of point 2 in (**a**).

Figure 4a shows the true label map delineated by visual interpretation using a 1:200,000 geological map of the study area and Google Earth Pro’s high-resolution images with field work (shown in Figure 5). It includes three plutons: Xiaerpu (left), Karamay (center), and Hongshan (right). Figure 4b shows the results extracted by the Otsu method without additional parameter settings. In the iterations of the Sauvola algorithm, the parameter settings of R = 128, k = −0.1, and window size of 300 performed well in this study area. To maintain comparability, the BR-ISauvola algorithm should use the same values for the shared parameters. Their extraction results are shown in Figure 4c,d. We can see from the figure that the Otsu method exhibits severe under-segmentation for the Xiaerpu pluton and over-segmentation for the Hongshan pluton. Both the Sauvola and BR-ISauvola methods successfully extract most of the granites. In the case of the Xiaerpu and Hongshan plutons, the former exhibits significant fragmentation along the boundaries and some over-segmentation, while the latter demonstrates lighter over-segmentation with neater boundaries and fewer fragmented areas. Additionally, both methods show slight under-segmentation for the Xiaerpu pluton.

Further precision analysis of the extraction results in shows that the accuracy and recall of the Otsu method are the lowest, and the F1 score is only 71.49% (shown in Table 3). The Sauvola algorithm has a relatively low omission error but a significantly higher commission error than BR-ISauvola because the lower threshold calculation reduces the omission error at the expense of the commission error. In contrast, the proposed BR-ISauvola method considers local brightness heterogeneity, substantially reducing the commission error without significantly affecting the omission error. Consequently, it achieves the highest F1 score of 82.11%. This indicates that SDGSAT-1 TIS data can serve as viable data for granite extraction.

## 4. Discussion

Numerous granite bodies have been developed in the West Junggar, which have been proven to have good potential for rare metal mineralization, and are one of the important target geological bodies for finding rare metal deposits in the Central Asian orogenic belt [31]. Currently, most of the data used in remote sensing to extract granite information are multispectral, hyperspectral, and high-resolution images; high costs of data and procedure are the common characteristics of these studies. In this study, the SDGSAT-1 TIS image, which provides high-resolution and wide-swath thermal infrared data, was used as a data source for granite extraction and different from conventional research. A methodology based on band ratio and image segmentation for extracting granite was proposed. The results demonstrated a high degree of conformity with the true label map, effectively delineating the three major plutons with an accuracy of more than 82%. This indicates the feasibility of the granite extraction method using SDGSAT-1 TIS data, and the results are important indications for geological surveys such as lithological mapping and mineral exploration in the area with harsh environments and limited data. It is noteworthy that all three methods exhibited omission errors in extracting the Xiaerpu pluton, where certain pixels were misclassified as background, possibly due to significant weathering and alteration causing changes in their spectral characteristics.

From a petrological and geochemical perspective, the granite origins in the Karamay region are similar but slightly different in time. The Hongshan and Karamay plutons were formed at about 330 Ma, characterized as quartz–alkali feldspar granites with high quartz and orthoclase content. In contrast, the Xiaerpu pluton was formed at about 298 Ma, representing a diorite–monzonite granite with a significant presence of ferromagnesian minerals [32,33,34,35]. The abundance of iron–magnesium minerals causes a relatively weak response in SDGSAT-1 TIS, leading to partial omission errors due to the low sensitivity of the sensor in the direction of longer wavelengths [22]. To address this limitation, we want to further explore the application of SDGSAT series satellite data and integrate other datasets such as multispectral and hyperspectral data for a more accurate study of granite extraction.

## 5. Conclusions

This paper proposes a granite extraction method based on SDGSAT-1 TIS data, and the main conclusions are as follows:

(1) SDGSAT-1 TIS data can serve as a valuable data source for extracting granite.

The high spatial resolution and three-band coverage of SDGSAT-1 TIS data make it a useful data source for granite extraction. In the case of granite extraction in the Karamay of Xinjiang, all three methods demonstrated an accuracy of over 70%, suggesting the potential of SDGSAT-1 TIS data for extracting geological information such as granite. SDGSAT-1 TIS images have high spatial resolution and three bands with a wide swath. The accuracy of the three methods in granite extraction in the Karamay area, for example, reaches more than 70%, which indicates to a certain extent that SDGSAT-1 TIS data can be used as a useful source of data for the extraction of geologic information such as granite.

(2) The BR-ISauvola method proposed in this paper is applicable to granite extraction.

In the BR-ISauvola method, the F1 score of the extraction results reaches 82.11%, which is 0.34% and 10.62% higher than that of Otsu and Sauvola, respectively, indicating that the method proposed in this paper is feasible in practical applications, especially in the absence of data sources to achieve fast and efficient extraction of granite classes over large areas.

Results of the three methods indicate that SDGSAT-1 TIS data may have a slight limitation in spectral response to granites with higher alkaline mineral content, such as iron–magnesium minerals. This insight provides a reference for the future development of SDGSAT-1 and is one of the directions for our future research.

## Figures and Tables

**Table 1:** Characteristics of SDGSAT-1 TIS bands.

SDGSAT-1 Thermal Infrared Spectrometer	
	B1: 8~10.5
Wavelength (μm)	B2: 10.3~11.3
	B3: 11.5~12.5
Width (km)	300
Spatial resolution (m)	30

**Table 2:** Radiometric calibration coefficients for SDGSAT-1 TIS bands.

Band	Gain	Bias
1	0.003947	0.167126
2	0.003946	0.124622
3	0.005329	0.222530

**Table 3:** Accuracy evaluation of different methods.

Methods	Omission Error%	Commission Error%	Precision%	Recall%	F1 Score%
Otsu	36.49	18.24	81.76	63.51	71.49
Sauvola	22.72	13.19	86.81	77.28	81.77
BR-ISauvola	24.95	8.76	91.24	75.05	82.11

## Data Availability

Data are obtained on 12 November 2023 by registering on the SDGSAT-1 Open Science Program website (see https://www.sdgsat.ac.cn/) to request access to SDGSAT-1 data.
